# Blood Pressure Complexity Discriminates Pathological Beat‐to‐Beat Variability as a Marker of Vascular Aging

**DOI:** 10.1161/JAHA.121.022865

**Published:** 2022-01-19

**Authors:** Yun‐Kai Lee, Sara Mazzucco, Peter M. Rothwell, Stephen J. Payne, Alastair J. S. Webb

**Affiliations:** ^1^ Institute of Biomedical Engineering Department of Engineering Science University of Oxford UK; ^2^ Wolfson Centre for Prevention of Stroke and Dementia Nuffield Department of Clinical Neurosciences John Radcliffe Hospital University of Oxford UK

**Keywords:** arterial stiffness, baroreflex sensitivity, blood pressure variability, complexity, heart rate variability, stroke, transient ischemic attack, Hypertension

## Abstract

**Background:**

Beat‐to‐beat blood pressure variability (BPV) is associated with an increased risk of stroke but can be driven by both healthy physiological processes and failure of compensatory mechanisms. Blood pressure (BP) complexity measures structured, organized variations in BP, as opposed to random fluctuations, and its reduction may therefore identify pathological beat‐to‐beat BPV.

**Methods and Results:**

In the prospective, population‐based OXVASC (Oxford Vascular Study) Phenotyped Cohort with transient ischemic attack or minor stroke, patients underwent at least 5 minutes of noninvasive beat‐to‐beat monitoring of BP (Finometer) and ECG to derive the following: BPV (coefficient of variation) and complexity (modified multiscale entropy) of systolic BP and diastolic BP, heart rate variability (SD of R‐R intervals), and baroreflex sensitivity (BRS; Welch's method), in low‐ (0.04–0.15 Hz) and high‐frequency (0.15–0.4 Hz) bands. Associations between BPV or BP complexity with autonomic indexes and arterial stiffness were determined (linear regression), unadjusted, and adjusted for age, sex, and cardiovascular risk factors. In 908 consecutive, consenting patients, BP complexity was inversely correlated with BPV coefficient of variation (*P*<0.001) and was similarly reduced in patients with hypertension or diabetes (*P*<0.001). However, although BPV coefficient of variation had a U‐shaped relationship with age, BP complexity fell systematically across age quintiles (quintile 1: 15.1 [14.0–16.1] versus quintile 5: 13.8 [12.4–15.1]) and was correlated with markers of autonomic dysfunction (heart rate variability SD of R‐R intervals: *r* = 0.20; BRS low frequency: 0.19; BRS high frequency: 0.26) and arterial stiffness (pulse wave velocity: −0.21; all *P*<0.001), even after adjustment for clinical variables (heart rate variability SD of R‐R intervals: 0.12; BRS low frequency and BRS high frequency: 0.13 and 0.17; and pulse wave velocity: −0.07; all *P*<0.05).

**Conclusions:**

Loss of BP complexity discriminates BPV because of pathological failure of compensatory mechanisms and may represent a less confounded and potentially modifiable risk factor for stroke.

**Nonstandard Abbreviations and Acronyms**
BPV: blood pressure variability
BRS: baroreflex sensitivity
CV: coefficient of variation
DBP: diastolic blood pressure
HF: high frequency
HRV: heart rate variability
LF: low frequency
modMSE: modified multiscale entropy
OXVASC: Oxford Vascular Study
PP: pulse pressure
SBP: systolic blood pressure

**Clinical Perspective**

**What Is New?**

- Beat‐to‐beat blood pressure (BP) variability shows a U‐shaped relationship with age.
- “Complexity” of BP, representing structured as opposed to random variability, is more linearly and inversely associated with age.
- Reduced complexity of BP is associated with major cardiovascular risk factors and markers of autonomic dysfunction and vascular aging.

**What Are the Clinical Implications?**

- Increased BP variability can be driven by either intact physiological processes in the young or pathological failure of compensatory mechanisms that are more common in older patients.
- The nonlinear relationship between BP variability and age confounds understanding of the relationship between underlying mechanisms, risk of stroke, and response to treatment.
- Complexity discriminates organized and structured variations in BP from increased BP variability attributable to autonomic dysfunction and vascular aging, and may be a more precise marker of future modifiable vascular risk.

Uncontrolled high blood pressure (BP) increases the risk of stroke and all cardiovascular events,^1^ whereas visit‐to‐visit,^2^, ^3^ day‐to‐day,^4^ and beat‐to‐beat^5^ BP variability (BPV) predict the risk of recurrent stroke, all cardiovascular events, cognitive impairment,^6^ and death. However, short‐term, beat‐to‐beat BPV, estimated by conventional statistical estimates (eg, SD and coefficient of variations [CV]), shows a U‐shaped relationship with age,^7^ likely reflecting higher BPV attributable to intact autonomically driven fluctuation of BPV in younger people but also reflecting failure of compensatory mechanisms^8^, ^9^ caused by aging and hypertension. The prognostic value of short‐term, beat‐to‐beat BPV is therefore likely to represent both impaired autonomic function, as seen after acute ischemic stroke,^10^ intracerebral hemorrhage,^11^ and subarachnoid hemorrhage,^12^ as well as vascular aging (arterial stiffness and pulse pressure [PP]).^13^ However, its validity across all patient groups may be confounded by increased short‐term, beat‐to‐beat BPV attributable to intact physiological processes^9^, ^14^, ^15^ in the young that manifests as a more physiologically organized fluctuation within the physiological signals.

Entropy‐based complexity analysis of BP measures variations in BP^16^, ^17^, ^18^ that are related to nearby variations at different time scales, thereby reflecting structured or organized variability (ie, complex variability or complexity), as opposed to random fluctuations.^14^ It has been proposed as a potential marker of physiological adaptability and intact compensatory mechanisms.^19^, ^20^ It is derived from the degree of nonlinear self‐similarity of signals, hence reflecting “organized” variability in BP^21^, ^22^, ^23^; and is more resistant to nonlinearity and nonstationarity of beat‐to‐beat recordings that undermine the accuracy and the validity of conventional analytic approaches.^24^

Reduced complexity of physiological signals has been associated with aging,^9^, ^15^ increased risk and greater frailty during cardiac surgery^21^, ^22^, ^25^ and after extracorporeal perfusion,^26^, ^27^ and worse outcomes after major ischemic stroke,^28^, ^29^ hemorrhagic stroke,^30^, ^31^ and traumatic brain injury.^32^, ^33^ However, the physiological and clinical validity of BP complexity has not been determined, because of the lack of proper physiological definition, short recording length of measurements, and limited study sizes.

Therefore, we hypothesised that (1) beat‐to‐beat BPV has a similar physiological basis to heart rate variability, as well as longer forms of BP variability, and therefore BPV may be increased in both healthy patients and patients with age‐related, vascular dysfunction,^14^, ^15^ such as impaired autonomic function or arterial stiffening; and (2) BP complexity potentially reflects organized physiological processes, and therefore may discriminate physiological from pathological forms of BPV. We therefore determined the associations between short‐term beat‐to‐beat BP complexity with measures of BPV, clinical characteristics, and markers of autonomic dysfunction and vascular aging in a large, population‐based cohort with recent transient ischemic attack (TIA) or minor stroke.

## Methods

Access to the data that support the findings of this study will be considered on application to the chief investigator on reasonable request. Please contact Professor Peter Rothwell for further information (peter.rothwell@ndcn.ox.ac.uk).

### Study Population and Research Ethical Approval

Consecutive, consenting patients within 6 weeks of a TIA or minor stroke (defined as National Institutes of Health Stroke Scale score, <5 ) were recruited between September 2010 and November 2019, as part of the Phenotyped Cohort of the OXVASC (Oxford Vascular Study).^34^, ^35^, ^36^ Participants were recruited at the OXVASC daily emergency clinic, either following a referral after attendance at the emergency department or after direct referral from primary care, usually within 24 hours. The OXVASC population consists of >92 000 individuals registered with about 100 primary care physicians in Oxfordshire, UK.^7^, ^34^, ^35^, ^36^

All consenting patients underwent a standardized medical history and examination, ECG, blood tests, magnetic resonance imaging of brain and contrast‐enhanced magnetic resonance angiography (or computed tomography of brain and either carotid Doppler ultrasound or computed tomography angiogram), an echocardiogram, and 5‐day ambulatory cardiac monitoring. All patients were reviewed by a study physician, the diagnosis was verified by the senior study neurologist (P.M.R.), and patients were followed up face‐to‐face for up to 10 years.^7^, ^34^, ^35^, ^36^ Consenting patients underwent a physiological assessment at the 1‐month follow‐up visit. Participants were excluded from this analysis if they were aged <18 years, cognitively impaired (Mini‐Mental State Examination score, <23), or pregnant; had atrial fibrillation, active cancer, autonomic failure, a recent myocardial infarction, unstable angina, heart failure (New York Heart Association 3–4 or ejection fraction <40%), or untreated bilateral carotid stenosis (>70%). OXVASC is approved by the Oxfordshire Research Ethics Committee A.^34^, ^35^, ^36^

### Data Acquisition

As part of the phenotyped cohort, a routine prospective cardiovascular physiological assessment is performed at the 1‐month follow‐up visit in a quiet, dimly lit, temperature‐controlled room (21 °C –23 °C). Continuous 3‐lead ECG and noninvasive finger arterial BP (Finometer MIDI; Finapres Medical Systems, the Netherlands) were measured over at least 5 minutes, and up to 10 minutes, at 200 Hz during supine conditions via a Powerlab 8/35 (ADInstruments) software, preferentially measured from the middle phalanx of the middle finger of the nondominant arm when possible.^5^, ^7^, ^35^, ^36^

Consecutive R‐R intervals of the ECG waveforms and beat‐to‐beat averages of systolic (SBP) and diastolic (DBP) components of BP were automatically derived and median filtered, with quadratic interpolation of the peak of the QRS complex and linear interpolation across ectopic beats.^4^ All recordings were visually reviewed by an experienced operator (A.J.S.W.) for quality assessment (3, optimal; 2, adequate for analysis; 1, severe artefacts; and 0, no data) blind to clinical information, based on the presence of artefacts or drift in the baseline, as previously described.^7^, ^37^ Only the recordings with optimal and adequate quality were included in the analysis.^7^, ^36^, ^37^

### Analysis

#### Complexity of BP

Beat‐to‐beat SBP and DBP signals were detrended by linear regression. Because of the relatively short recording length of measurements, complexity of beat‐to‐beat BP was determined by the modified multiscale entropy^38^ (modMSE), which was specifically developed for shorter‐length time series by Wu et al.^38^ The modMSE calculates the sample entropy^39^ across multiple time scales to quantify the degree of irregularity of the signal with a moving‐average procedure to address the inaccurate entropy estimates caused by shortened data length in the conventional multiscale entropy algorithm.^19^, ^20^ By plotting sample entropy against the scale factor, the modMSE curve can be obtained.^38^ We set the scale from 1 to 10 and determined the complexity index by integrating the area under the modMSE curve, as described in previous studies.^27^, ^28^, ^29^, ^31^, ^32^ (Details of calculations are described in Data S1, whereas we also refer the readers to the study by Wu et al^38^ for detailed derivations of modMSE).

#### Derivations of Indexes of Autonomic Function and Vascular Aging

Heart rate variability (HRV) was estimated by the SD of R‐R intervals and root mean square of the successive beat‐to‐beat difference.^28^, ^30^ Baroreflex sensitivity (BRS) was calculated from the mean values in the defined frequency regions of sympathetic and parasympathetic activations of low‐frequency (LF; 0.04–0.15 Hz) and high‐frequency (HF; 0.15–0.4 Hz) bands, respectively,^28^, ^30^ using the transfer function between SBP and pulse interval^4^ (Welch’s methods; by the CARNet transfer function script [http://www.car‐net.org/]) with criterion of default coherence threshold as set in the script, based on the 95% CI of the null hypothesis of no relationship between input and out signals.^40^ The power spectrum densities of SBP and DBP were also determined by calculating the area in the same defined LF and HF regions, with derivation of LF/HF ratio of sympathetic‐to‐parasympathetic balance.^28^, ^30^

Aortic arterial stiffness was estimated by carotid‐femoral pulse wave velocity, measured by applanation tonometry^41^ (Sphygmocor; AtCor Medical, Sydney, Australia). PP was calculated as the difference between SBP and DBP ^3^ (PP=SBP‐DBP), and the systolic and diastolic BPV were calculated as the coefficient of variation (CV=100%×(SD/mean)) of the continuous beat‐to‐beat BP monitoring (SBP‐CV and DBP‐CV, respectively).^5^, ^35^, ^36^

### Statistical Analysis

The distributions of continuous variables were assessed by histograms and tested for normality (Shapiro‐Wilk). Clinical characteristics were compared by the χ^2^ test for categorical variables and ANOVA for continuous variables. Associations between BP complexity with HRV, BRS, and continuous clinical characteristics were assessed by linear regression with a log transformation to normalize the data and improve validity of the regression model, both unadjusted and adjusted for clinical characteristics, reported as partial correlation coefficients (*r* values), stratified by sex, age in quintiles (<54.2, 54.2–64.7, 64.7–71.4, 71.4–77.7, and >77.7), and hypertension or diabetes.

For all analyses, a value of *P<*0.05 was considered to be statistically significant. All analysis was performed in Microsoft Excel, Matlab r2017, and R.

## Results

A total of 959 of 1013 (95%) eligible, consenting patients had at least adequate beat‐to‐beat BP recordings, of whom 51 (5%) had inadequate recording quality or atrial fibrillation during testing, 3 (0.3%) had poor quality ECG recordings, and 93 (9.7%) did not have pulse wave velocity assessed because of technical limitations, such as body habitus or significant carotid stenosis (Table 1).

**Table 1:** Characteristics of Study Population, Stratified by Quintiles of Age

Characteristic	All*	Quintile 1	Quintile 2	Quintile 3	Quintile 4	Quintile 5	*P* value
No.	908	182	181	182	181	182	
Age, mean (SD), y	66.1 (13.3)	45.6 (8.1)	59.7 (3.0)	68.4 (2.0)	74.5 (1.8)	82.1 (3.4)	<0.001
Women, n (%)	411 (45.3)	66 (36.3)	82 (45.3)	86 (47.3)	92 (50.8)	85 (46.7)	0.07
Hypertension, n (%)	442 (48.7)	40 (22.0)	73 (40.3)	96 (52.7)	109 (60.2)	124 (68.1)	<0.001
Diabetes, n (%)	107 (11.8)	19 (10.4)	22 (12.2)	18 (9.9)	24 (13.3)	24 (13.2)	0.79
Current smoking, n (%)	152 (16.7)	60 (33.0)	43 (23.8)	28 (15.4)	12 (6.6)	9 (4.9)	<0.001
Antihypertensive agents, n (%)	670 (73.8)	98 (53.8)	117 (64.6)	148 (81.3)	153 (84.5)	154 (84.6)	<0.001
BMI, mean (SD), kg/m^2^	27.2 (5.3)	27.9 (6.6)	28.5 (5.5)	26.7 (4.9)	26.5 (4.2)	26.2 (4.5)	<0.001
SBP, mean (SD), mm Hg	125.4 (18.0)	119.3 (15.8)	121.0 (13.8)	127.6 (18.3)	128.9 (18.5)	130.3 (20.1)	<0.001
DBP, mean (SD), mm Hg	68.8 (10.0)	70.8 (10.5)	70.9 (9.2)	68.7 (10.3)	68.0 (9.4)	65.5 (9.9)	<0.001
PP, mean (SD), mm Hg	56.6 (14.8)	48.5 (11.8)	50.1 (11.2)	58.9 (13.5)	61.0 (13.9)	64.7 (16.3)	<0.001
PWV, mean (SD), m/s	9.6 (2.7)	7.5 (1.7)	8.5 (1.8)	9.7 (2.1)	10.6 (2.5)	12.1 (3.0)	<0.001

Frequency, number (percentage), or mean (SD) are reported. *P* values are given for ANOVA for continuous variables and χ^2^ test for categorical variables. Quintile 1 indicates <54.2 years; quintile 2, 54.2 to 64.7 years; quintile 3, 64.7 to 71.4 years; quintile 4, 71.4 to 77.7 years; and quintile 5, >77.7 years. BMI indicates body mass index; DBP, diastolic blood pressure; PP, pulse pressure; PWV, pulse wave velocity; and SBP, systolic blood pressure.

* One patient with missing diagnosis of all comorbidities; 93 patients did not have PWV assessed; 2 patients with missing information of antihypertensive agents; and 63 patients with missing information of BMI.

### Distributions of Beat‐to‐Beat BPV and BP Complexity

The distributions of BPV of SBP‐CV and DBP‐CV (medians [interquartile intervals]: 4.7 [3.5–6.6] and 4.6 [3.3–6.6], respectively) were strongly positively skewed and nonnormal (*P*‐normality <0.001; Table S1). Distributions of complexity of SBP and DBP (medians [interquartile intervals]: 14.5 [13.2–15.7] and 14.7 [13.4–15.7], respectively) were less negatively skewed (Table S1 and Figure S1), and were more normally distributed than BPV, even when stratified by quintiles of age (Table S1). BPV and complexity remained skewed when stratified by sex, but complexity of both SBP and DBP was largely normally distributed and less skewed compared with BPV, even in the upper quintiles of age (Tables S2 and S3).

### Associations of Beat‐to‐Beat BPV and BP Complexity With Age and Major Risk Factors

**Figure 1:**
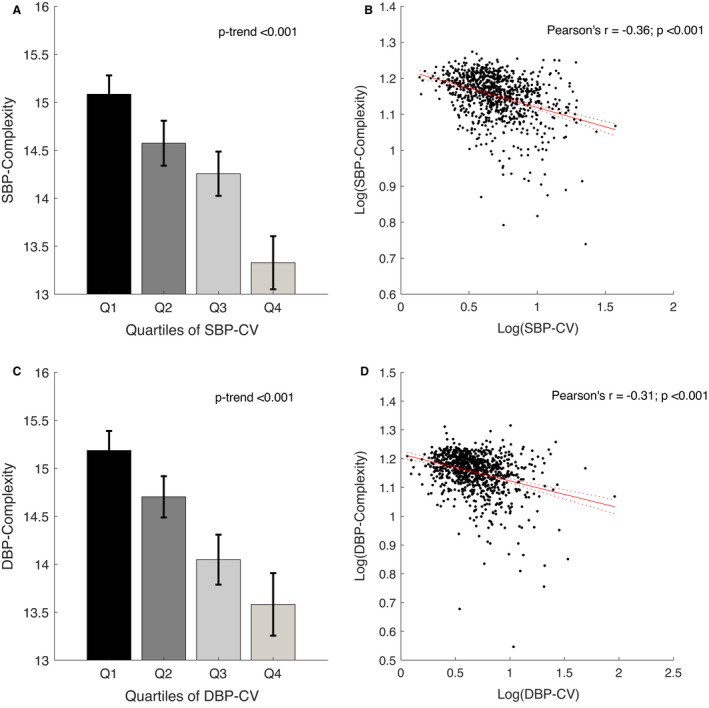
Correlations of blood pressure complexity with measures of blood pressure variability. Systolic blood pressure (SBP) complexity vs SBP coefficient of variation (CV) (**A** and **B**) and diastolic blood pressure (DBP) complexity vs DBP‐CV (**C** and **D**), stratified by quartiles and for the linear regression with a log transformation of both indexes to normalize the data and improve validity of the regression model. Values of *P*‐trend are given for linear regression. Data are presented as mean with 95% CI and regression line with 95% CI.

**Figure 2:**
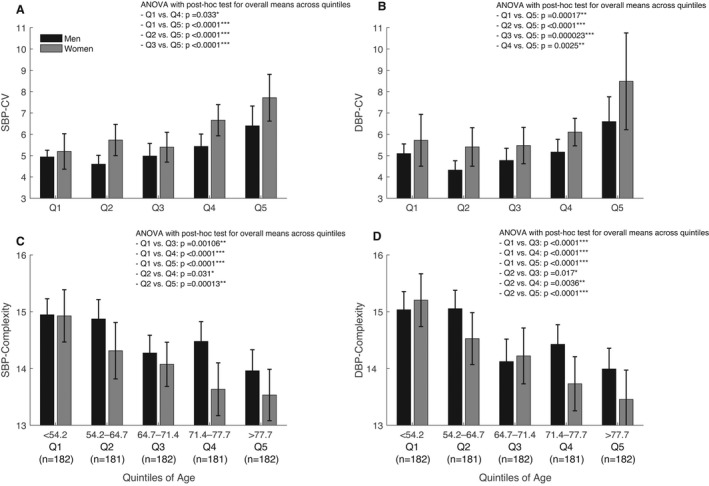
Changes of beat‐to‐beat blood pressure variability and complexity, stratified by sex and by quintiles (Qs) of age. **A**, Systolic blood pressure (SBP) coefficient of variation (CV). **B**, Diastolic blood pressure (DBP) CV. **C**, Complexity of SBP. **D**, Complexity of DBP. *P* values are given for ANOVA with post hoc analysis (Tukey test) for comparisons among the overall means across the 5 quintiles. Only the results that reach the statistically significant level are presented. Data are presented as mean with 95% CI. *P<0.05, **P<0.01, and ***P<0.001.

Complexity of BP was negatively correlated with measures of BPV (SBP complexity versus SBP‐CV and DBP complexity versus DBP‐CV: *r*=−0.36 and *r*=−0.31, respectively; both *P*<0.001), with a linearly falling trend when stratified by quartiles (Figure 1; both *P*‐trend <0.001). SBP‐CV and DBP‐CV were nonlinearly related to age, with greater BPV in the bottom quintile compared with the second and third quintiles, followed by a progressive increase in upper quintiles (Figure 2A and 2B). However, complexity of both SBP and DBP was more linearly reduced across quintiles of age, with a more pronounced reduction in women than men (Figure 2C and 2D; Figure S2). This falling complexity of both SBP and DBP across quintiles of age persisted when stratified by events of TIA and stroke (Figure S3).

Patients with hypertension or diabetes had higher SBP‐CV and DBP‐CV, with the highest BPV in patients with both comorbidities (Table 2), but with no interaction between hypertension and diabetes even after adjustment for age, sex, cardiovascular risk factors, and smoking (adjusted *P*=0.95 and *P*=0.89, respectively; Table 2). BP complexity was lower in patients with hypertension and diabetes, and lowest in patients with both comorbidities (Table 2), with no significant interaction between hypertension and diabetes, including after adjustment for clinical variables (complexity of SBP and DBP: *P*=0.49 and *P*=0.4, respectively; Table 2).

**Table 2:** Values and Associations of BPV and Complexity With Pathology of Major Cardiovascular Risk Factors

Variable	Absolute values			
	SBP‐CV, %	DBP‐CV, %	SBP‐complexity	DBP‐complexity
Nonhypertension vs hypertension	5.5 (5.2 to 5.8) vs 5.9 (5.5 to 6.2)	5.4 (5.1 to 5.8) vs 5.9 (5.4 to 6.5)	14.6 (14.4 to 14.8) vs 14.0 (13.8 to 14.2)	14.7 (14.6 to 14.9) vs 14.0 (13.8 to 14.2)
Nondiabetes vs diabetes	5.6 (5.4 to 5.8) vs 6.4 (5.6 to 7.2)	5.5 (5.2 to 5.8) vs 7.0 (5.1 to 8.8)	14.4 (14.3 to 14.5) vs 13.6 (13.3 to 14.0)	14.5 (14.3 to 14.6) vs 13.7 (13.3 to 14.1)
Combined hypertension and diabetes	6.5 (5.5 to 7.6)	7.3 (4.9 to 9.6)	13.4 (13.0 to 13.1)	13.6 (13.1 to 14.0)

	Regression analysis							
	SBP‐CV, %		DBP‐CV, %		SBP‐complexity		DBP‐complexity	
	B (95% CI)	*P* value	B (95% CI)	*P* value	B (95% CI)	*P* value	B (95% CI)	*P* value
Unadjusted								
Hypertension	0.01 (−0.05 to 0.08)	0.67	0.03 (−0.05 to 0.10)	0.46	−0.03 (−0.05 to −0.01)*	0.002*	−0.05 (−0.07 to −0.03)*	<0.001*
Diabetes	0.11 (−0.09 to 0.31)	0.27	0.10 (−0.12 to 0.31)	0.37	−0.03 (−0.09 to −0.03)	0.31	−0.06 (−0.13 to −0.001)*	0.048*
Hypertension×diabetes	−0.005 (−0.23 to 0.23)	0.97	−0.02 (−0.27 to 0.24)	0.90	−0.02 (−0.09 to 0.05)	0.55	0.03 (−0.05 to 0.10)	0.46
Adjusted (age+sex)								
Hypertension	−1.24 (−1.41 to −1.07)*	<0.001*	−1.27 (−1.46 to −1.08)*	<0.001*	−2.82 (−2.87 to −2.77)*	<0.001*	−2.86 (−2.92 to −2.81)*	<0.001*
Diabetes	−1.08 (−1.35 to −0.81)*	<0.001*	−1.15 (−1.45 to −0.85)*	<0.001*	−2.84 (−2.92 to −2.76)*	<0.001*	−2.91 (−2.99 to −2.82)*	<0.001*
Hypertension×diabetes	−0.02 (−0.24 to 0.21)	0.89	−0.02 (−0.27 to 0.23)	0.87	−0.02 (−0.08 to 0.05)	0.63	0.03 (−0.04 to 0.11)	0.36
1+hypertension+diabetes	1.22 (1.05 to 1.38)*	<0.001*	1.27 (1.09 to 1.45)*	<0.001*	2.80 (2.75 to 2.85)*	<0.001*	2.83 (2.78 to 2.88)*	<0.001*
Age	0.005 (0.003 to 0.008)*	<0.001*	0.003 (0.001 to 0.006)*	0.014*	−0.002 (−0.003 to −0.001)*	<0.001*	−0.002 (−0.003 to −0.001)*	<0.001*
Sex	0.11 (0.05 to 0.18)*	<0.001*	0.11 (0.04 to 0.18)*	0.0018*	−0.04 (−0.06 to −0.02)*	<0.001*	−0.03 (−0.05 to −0.01)*	0.002*
Adjusted (age+sex+smoking)								
Hypertension	−1.19 (−1.37 to −1.0)*	<0.001*	−1.24 (−1.44 to −1.04)*	<0.001*	−2.86 (−2.91 to −2.81)*	<0.001*	−2.88 (−2.94 to −2.82)*	<0.001*
Diabetes	−1.03 (−1.30 to −0.75)*	<0.001*	−1.12 (−1.43 to −0.82)*	<0.001*	−2.88 (−2.96 to −2.80)*	<0.001*	−2.92 (−3.01 to −2.84)*	<0.001*
Hypertension×diabetes	−0.007 (−0.23 to 0.22)	0.95	−0.02 (−0.27 to 0.23)	0.89	−0.02 (−0.09 to 0.04)	0.49	0.03 (−0.04 to 0.10)	0.40
1+Hypertension+diabetes	1.16 (0.98 to 1.33)*	<0.001*	1.24 (1.05 to 1.44)*	<0.001*	2.85 (2.79 to 2.90)*	<0.001*	2.85 (2.79 to 2.91)*	<0.001*
Age	0.006 (0.003 to 0.008)*	<0.001*	0.004 (0.001 to 0.007)*	0.01*	−0.002 (−0.003 to −0.002)*	<0.001*	−0.002 (−0.003 to −0.002)*	<0.001*
Sex	0.12 (0.05 to 0.18)*	0.0003*	0.12 (0.04 to 0.19)*	0.0016*	−0.04 (−0.06 to −0.02)*	<0.001*	−0.03 (−0.05 to −0.01)*	0.0015*
Current smoking	0.09 (−0.003 to 0.18)	0.059	0.04 (−0.06 to 0.14)	0.43	−0.06 (−0.09 to −0.04)*	<0.001*	−0.03 (−0.06 to −0.003)*	0.047*

Data are presented as mean with 95% CIs. Associations were determined by general linear models with a log transformation. B (95% CI) indicates the unstandardized regression coefficients with 95% CIs; hypertension×diabetes, the interaction effects between hypertension and diabetes; 1+hypertension+diabetes, the independent term of hypertension and diabetes; adjusted (age+sex), adjusted for age and sex; and adjusted (age+sex+smoking), adjusted for age, sex, and smoking habit. BPV indicates blood pressure variability; CV, coefficient of variation; DBP, diastolic blood pressure; and SBP, systolic blood pressure.

* Statistically significant.

### Associations of BP Complexity With Autonomic Functions and Vascular Aging

**Figure 3:**
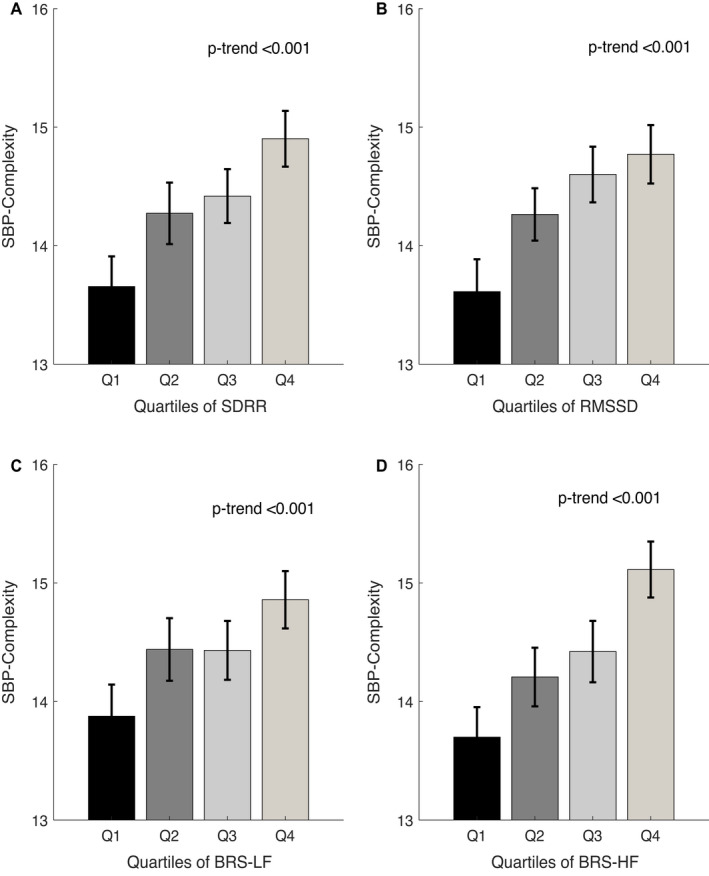
Values of complexity of systolic blood pressure (SBP), stratified by quartiles (Qs) of parameters of SD of R‐R intervals (SDRR) (**A**), root mean square of the successive beat‐to‐beat difference (RMSSD) of R‐R intervals (**B**), and baroreflex sensitivity (BRS) in low‐frequency (LF) (**C**) and high‐frequency (HF) (**D**) bands. Three invalid quality of ECG recordings and those who do not meet the statistical criterion of BRS coherence were not included. Values of *P*‐trend are given for linear regression. Data are presented as mean with 95% CI.

**Figure 4:**
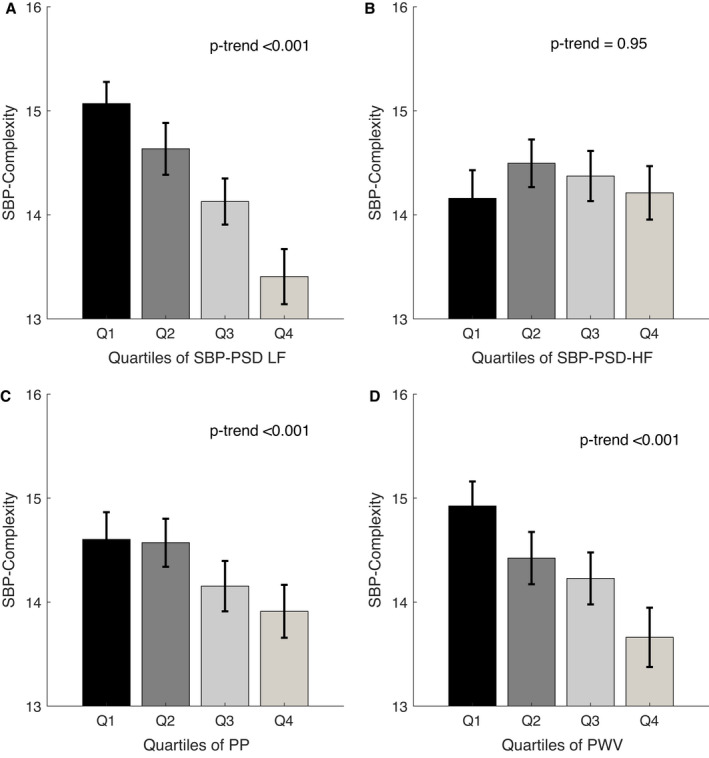
Values of complexity of systolic blood pressure (SBP), stratified by quartiles (Qs) of parameters of SBP power spectrum density (PSD) in low‐frequency (LF) (**A**) and high‐frequency (HF) (**B**) bands; and indexes of vascular aging of pulse pressure (PP) (**C**) and pulse wave velocity (PWV) (**D**). Patients (n=93) who did not have PWV assessed were not included. Values of *P*‐trend are given for linear regression. Data are presented as mean with 95% CI.

Greater complexity of SBP and DBP was correlated with impaired autonomic function, with a positive association with both SD of R‐R intervals and root mean square of the successive beat‐to‐beat difference (SBP complexity versus SD of R‐R intervals and root mean square of the successive beat‐to‐beat difference: adjusted *r*=0.12 and *r*=0.16, respectively), and a linear trend across quartiles (Figure 3A and 3B; Table S4; Figure S4). Similarly, SBP complexity was correlated with BRS gain in both LF and HF domains (adjusted *r*=0.13 and *r*=0.17, respectively; all *P*<0.001), before and after adjustment for clinical variables (Table S4; Figure 3C and 3D; all *P*‐trend <0.001), with a consistent result of association of complexity of DBP with BRS (Figures S4 and S5; Table S4). Across the entire frequency spectrum of BRS gain and power spectrum density of BP, higher complexity of both SBP and DBP was associated with a greater BRS gain but a lower power spectrum density of BP and LF/HF ratio, with a more pronounced difference in lower‐frequency bands (Figures S4–S6; Figure 4A; *P*‐trend<0.001), implying a reduced sympathetic to parasympathetic autonomic balance (Figures S4–S6; Figure 4B; *P*‐trend=0.95).

There was an inverse correlation between complexity of BP and indexes of vascular aging. Complexity of SBP was inversely correlated with both PP and pulse wave velocity (unadjusted *r*=−0.14 and *r*=−0.21, respectively), with a linear trend across quartiles (Figure 4C and 4D; all *P*‐trend<0.001). However, there was no significant association between complexity and PP after adjustment for clinical variables (Table S4). Associations of complexity of DBP with PP and arterial stiffness were consistent with complexity of SBP (Figures S5 and S6; Table S4).

## Discussion

In this large, prospective, clinical cohort of high‐risk patients with TIA or minor stroke, BP complexity and beat‐to‐beat BP variability were both found to be strongly associated with a history of hypertension and diabetes, with increased arterial stiffness, autonomic dysfunction, and increasing age. However, BP complexity was at least as strongly associated as BPV, was more normally distributed, and had a negative linear association with age, as opposed to beat‐to‐beat BPV, which increased in the youngest patients, likely attributable to healthy, physiologically determined fluctuations in BP. As such, BP complexity appears to provide both a specific measure of pathologically determined BP variability and a marker of vascular aging and autonomic dysfunction.

Visit‐to‐visit, day‐to‐day, and beat‐to‐beat BPV predict the risk of recurrent stroke and cardiovascular events, independent of mean BP.^5^, ^7^ However, currently available methods of assessing BPV on beat‐to‐beat monitoring show a nonlinear relationship with age that is confounded by increased variations in BP in younger patients, likely attributable to physiologically determined rhythmic fluctuations in BP.^7^, ^8^ This is mixed with potentially pathological, random variations that are more common with increased age and that result in a strong positive skew to the distribution as patients become older.^7^ BP complexity measures organized and structured variations in BP across multiple time periods. Therefore, its consistent linear association with age^19^, ^20^ and with markers of vascular aging and autonomic dysfunction supports the hypothesis that “raw” BPV is confounded by a mixture of healthy and pathological forms of BPV, whereas BP complexity is potentially a direct measure of healthy BP and its control. Its loss with age and underlying cardiovascular pathology of hypertension^17^ and diabetes^16^ may therefore be a direct measure of failure of these compensatory mechanisms.^8^, ^42^

There are no data on the long‐term predictive value of complexity of BP for cardiovascular disease, and its association with the risk of stroke and dementia is unclear.^43^ However, markers of autonomic dysfunction and vascular aging predict major stroke,^44^ lacunar stroke, and cognitive impairment,^45^ whereas complexity of HRV,^19^, ^20^ cerebrovascular blood flow derived from near‐infrared spectroscopy‐derived signals,^31^ and intracranial pressure^32^ predict outcome following acute ischemic stroke,^28^, ^29^ intracerebral (supratentorial and intraventricular) hemorrhage,^30^, ^31^ and severe traumatic brain injury,^32^, ^33^ and during cardiac surgery.^22^, ^25^, ^46^ Recent advances have also reported that this loss of “structured variability” in BP is associated with higher grade of white matter lesions in older adults^47^ and with elevated long‐term risk of dementia.^43^ Furthermore, the systematic loss of complexity of BP with age is consistent with smaller studies reporting reduced complexity of HRV in elder subjects.^19^, ^20^ As such, BP complexity has the potential to be an unconfounded marker of failure of compensatory vascular mechanisms that can be measured with 5 minutes of beat‐to‐beat BP recording. It therefore can help to determine to what extent failure of compensatory mechanisms explains the resulting risk of cardiovascular events with age, hypertension, and cardiovascular disease. It is likely to be more specific than BPV, but it is as yet not clear to what extent it is more sensitive than BPV, or to what extent any associated explained risk may be independent or additive to classic markers of vascular aging, such as arterial stiffness or baroreceptor sensitivity.

### Study Limitations

There are several limitations to our study. First, all patients were assessed for TIA or minor stroke events, predominantly in older patients. Hence, the understanding of complexity of BP in other populations or those with major stroke remains unclear.^7^ However, BP variability and beat‐to‐beat BPV appear to be particularly associated with the risk of stroke and dementia, and this population is therefore ideal to determine the value of BP complexity for these outcomes.^5^, ^43^ Second, this cohort is focused on prognostic factors and prevention of recurrent stroke and cardiovascular events; a significant proportion of patients are commonly prescribed with multiple antihypertensive medications to control BP, according to the treatment guidelines.^35^ As such, we cannot exclude an interaction between effects of antihypertensive drugs and cardiovascular mechanisms, and the standardized treatment protocol with agents from multiple classes prevents reliable comparison of class‐specific differences. Furthermore, in this nonrandomized study, observational analyses of the role of different BP classes are prone to confounding. However, the reduced BP complexity in our study is consistent with many previous studies reporting its age‐related reductions in healthy subjects and in multiple pathophysiological conditions.^19^, ^20^ This therefore indicates the potential of BP complexity to discriminate potentially beneficial and harmful forms of BPV in future studies, such as the recently demonstrated sex difference in BPV in obesity^36^ (eg, body mass index), effects of antihypertensive drug classes on BPV, and its association with future risk and clinical outcome.^5^, ^43^ Further investigation will also be necessary to specifically determine the changes of BPV and complexity in patients with carotid lesions that may affect the baroreceptor functions, patients with a cardiac pacemaker, and those with atrial fibrillation as the irregular R‐R intervals reflect a different physiological basis for BPV and BP complexity, and multiple studies have reported a different trend of complexity in patients with atrial fibrillation.^19^, ^20^, ^28^ Third, although previous studies have shown the effects of locations of hematoma on complexity (of HRV),^30^ this population had had TIA or minor stroke, including a significant number with no acute diffusion‐weighted imaging lesion on magnetic resonance imaging, in whom the precise site of cerebrovascular ischemia is unknown. Therefore, we are unable to determine whether infarction site affects BP complexity in this population. However, this also means that it is unlikely that the cerebrovascular events themselves had a significant impact on complexity. Fourth, compared with the currently favored methods of assessing BPV, such as SD or CV,^7^ the derivation of entropy‐based complexity is relatively complex. It is therefore necessary to investigate the feasibility, validity, and reproducibility of complexity indexes, before its integration to analytical tools in real‐time bedside monitoring in clinical practice.

Finally, we have not yet investigated the prognostic significance of complexity of BP for the risk of recurrent stroke as more patient‐years of follow‐up will be required for a reliable estimation. However, to our knowledge, this is the first large study investigating short‐term beat‐to‐beat BP complexity and is the first in a population‐based cohort with TIA or minor stroke to demonstrate its association with measures of BPV, clinical characteristics, and multiple systematic physiological markers of autonomic dysfunction and vascular aging. Longer‐term follow‐up in this population will allow us to determine the prognostic significance of BP complexity, and its added utility compared with classic cardiovascular risk factors, beat‐to‐beat BPV, and markers of vascular aging.

## Conclusions

Loss of BP complexity has the potential to differentiate increased BPV attributable to intact physiological mechanisms from increased BPV attributable to pathological failure of compensatory mechanisms, providing physiological information beyond only a single derived parameter. This provides a robust foundation of its application for future epidemiological or clinical studies to assess its prognostic significance and potentially as a modifiable risk factor for future cardiovascular events and recurrent stroke and dementia.

## Sources of Funding

OXVASC (Oxford Vascular Study) is funded by the National Institute for Health Research (NIHR) Oxford Biomedical Research Centre, Wellcome Trust, Wolfson Foundation, British Heart Foundation, and the European Union Horizon 2020 programme (grant 666881; SVDs@target). Dr Rothwell is in receipt of an NIHR Senior Investigator award. Dr Webb is funded by a Wellcome Trust Clinical Research Development Fellowship (206589/Z/17/Z) and British Heart Foundation Project Grant (PG/16/38/32080). The views expressed are those of the authors and not necessarily those of the National Health Service, the NIHR, or the Department of Health.

## Disclosures

None.

## Supporting information

Data S1
Tables S1–S4
Figures S1–S6
