# Supplementary material for: Blood Pressure Complexity Discriminates Pathological Beat‐to‐Beat Variability as a Marker of Vascular Aging
Source: J Am Heart Assoc. 2022 Jan 19;11(3):e022865. doi: 10.1161/JAHA.121.022865 (PMC9238484; doi:10.1161/JAHA.121.022865)
Supplement: Supplementary file 1 — Data S1 Tables S1–S4 Figures S1–S6 [file JAH3-11-e022865-s001.pdf]

SUPPLEMENTAL MATERIAL

Data S1.

Supplemental Methods

A1. Modified multiscale entropy (ModMSE)

The code for ModMSE can be found in the Appendix in the original publication by Wu et al. 2013. *Physica A.* 2013. doi:10.1016/j.physa.2013.07.075.

Entropy has been proposed as an estimate to quantify the degree of irregularity (or randomness) of a signal, and sample entropy (SampEn) is one of the methods commonly used, originally proposed by Richman and Moorman in 2000 (*Am J Physiol Heart Circ Physiol*. 2000; 278:H2039-49. doi: 10.1152/ajpheart.2000.278.6.H2039).

Its calculation is based on the negative logarithm of the number of the occurrence of repeating patterns (match components) that have distance smaller than the tolerance in the signal (figure A1). Given the time-series data $\boldsymbol{S} = \{x_1, x_2, x_3, \dots x_N\}$, the SampEn first constructs the similarity index (i.e., the i th template vector) of length m, $X_m(i) = \{x_i, x_{i+1}, x_{i+2}, x_{i+3}, \dots x_{(i+m-1)}\}$, as well as match vector of length $(m+1)$, $X_{m+1}(i)$. Sample entropy can then be described and calculated as follows:

$$SampEn(\boldsymbol{S}, \mathrm{m}, r, N) = -\ln\left[\frac{A_i}{B_i}\right]$$

where parameters m represent the dimension of constructing the template vector pairs; r indicates the tolerance threshold; N is the length of the signal; A_i is the number of the matches (i.e., the template vector) of length $(m+1)$ that has a distance smaller than r times the standard deviation (SD) of the signal, expressed as:

$$d[X_{m+1}(i), X_{m+1}(j)] < (r \times SD\ of\ the\ signal)$$

and B_i is the number of the matches of length(m) that has a distance smaller than tolerance r times the SD of the signal:

$$d[X_m(i), X_m(j)] < (r \times SD\ of\ the\ signal)$$

Later, Govindan et al., 2007 *(Physica A 376; 158–164)* further modified the definition of the original SampEn and incorporated a time-delay in calculating the match template vectors, where the SampEn with time-delay can thus be expressed as:

$$SampEn = (\boldsymbol{S}, m, r, \delta)$$

where δ is the time-delay between the successive match components when constructing the match templates:

$$\boldsymbol{X}_i^m(\delta) = \{x_i, x_{i+\delta}, \dots x_{(m-1)\delta}\}$$

Similarly, the distances for each match components are calculated by deriving the number of matches in this modified version of SampEn (Wu et al. 2013), as determined by:

$$d_{ij}^m(\delta) = \left\|\boldsymbol{X}_i^m(\delta) - \boldsymbol{X}_j^m(\delta)\right\|_\infty, 1 \le i, j \le N - m\delta, j > i + \delta$$

whilst m and r are the same parameters used for the dimension vector and tolerance threshold respectively.

Costa et al., 2002, 2005 (*Phys Rev Let*. 2002; 89(6):068102) proposed an extended method, termed the multiscale entropy (MSE) method[19,20], to determine the complexity of the signal. The process of this ***conventional*** **MSE** is: (i) to coarse-grain the signal by averaging the neighbouring data-points with non-overlapping window by the scale factor (i.e., τ); and (ii) to calculate the SampEn of each coarse-grained time-series; and (iii) by plotting the SampEn against scale factor, the MSE curve can be obtained. The coarse-grained time-series, y_j, can be expressed as follows:

$$y_j^\tau = \frac{1}{\tau} \sum_{i=(j-1)\tau+1}^{j\tau} \boldsymbol{S}_i, \qquad 1 \le j \le \frac{N}{\tau}$$

where τ represents the scale factor and N is the data length. For both original SampEn and conventional MSE, a unity-delay was applied ($\delta = 1$)[38].

However, the coarse-graining process in the conventional MSE shortens the data length, which may result in inaccurate estimates, particularly in short-term time-series. In 2013, Wu et al., 2013[38] thus proposed the modified multiscale entropy (ModMSE). The modMSE applies the sample entropy with time-delay and replace the coarse-graining process in the conventional MSE algorithm with a moving-average procedure. The moving-averaged time-series at scale factor τ is therefore expressed as:

$$z_j^{\tau} = \frac{1}{\tau}\sum_{i=j}^{j+\tau-1} \boldsymbol{S}_i, \qquad 1 \le j \le N - \tau + 1$$

The size of the moving-average window is set for both the time-delay and the scale factor to overcome the limitation of shortened data length, and the ModMSE is expressed as follows:

$$ModMSE(\boldsymbol{S}, m, \tau, r) = SampEn(z^{\tau}, m, \delta = \tau, r)$$

as described previously (Wu et al., 2013). Similarly, by plotting sample entropy against the scale factor τ, the ModMSE curve can be obtained.

Figure A2 demonstrates the simulation of modMSE with short-term time series signals using length of 500 data points. In this study, we set the parameters of *r = 0.2, m = 2,* and the scale from 1 – 10, which are the commonly selected numbers with better statistical validity[24]; and the complexity index is defined as the integration of the area under the modMSE curves, as described in previous studies[28-32].

Figure A1. Schematic of calculations of the original sample entropy, as described in Richman et al. 2004 (*Methods Enzymol.* 2004; 384:172-84. doi: 10.1016/S0076-6879(04)84011-4) and references [19,20,32]. Parameter *r* is set for the threshold for the tolerance for accepting matches; m, the dimension parameter (m = 2 in this case). Solid circles and dash circles are the match templates of (m+1) and (m) dimensions, respectively.

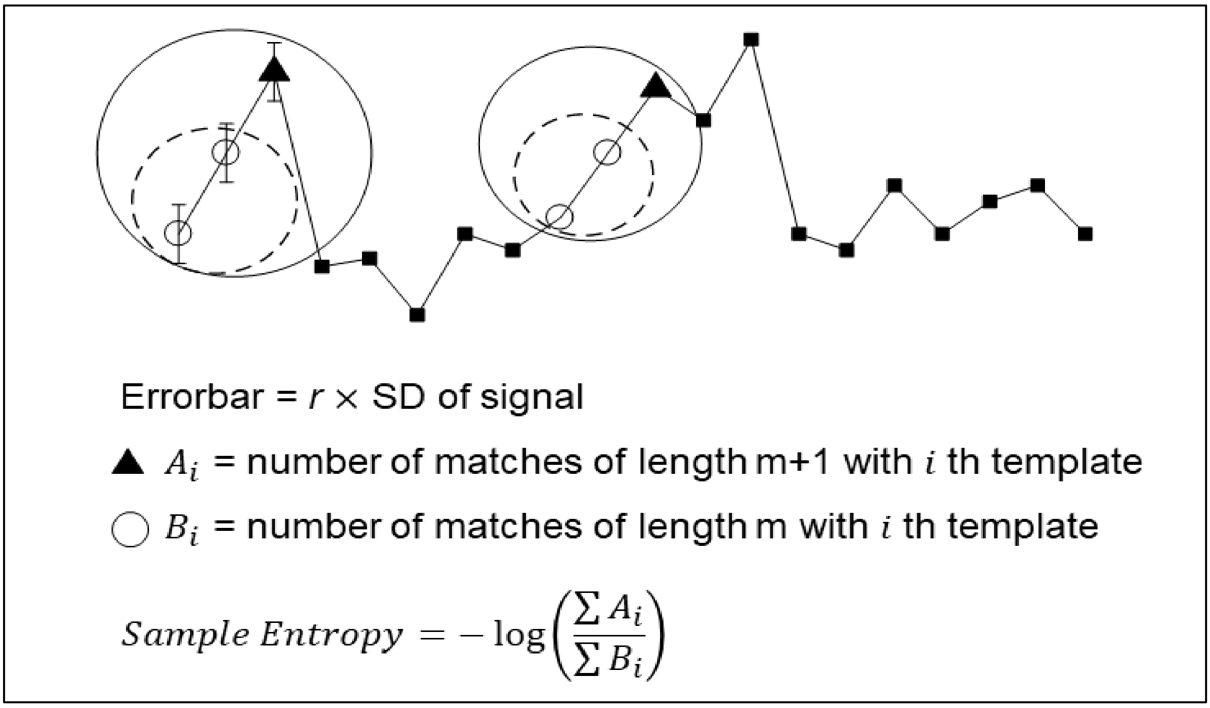

Figure A2. Simulations of modMSE analysis for short-term time series (500 data points). (A) and (B) are white noise (completely random noise) and pink noise (1/f noise), respectively; and (C) represents the modMSE curves of the averaged 10 independent simulations. Errorbars are presented as mean ± standard error of the mean (SEM).

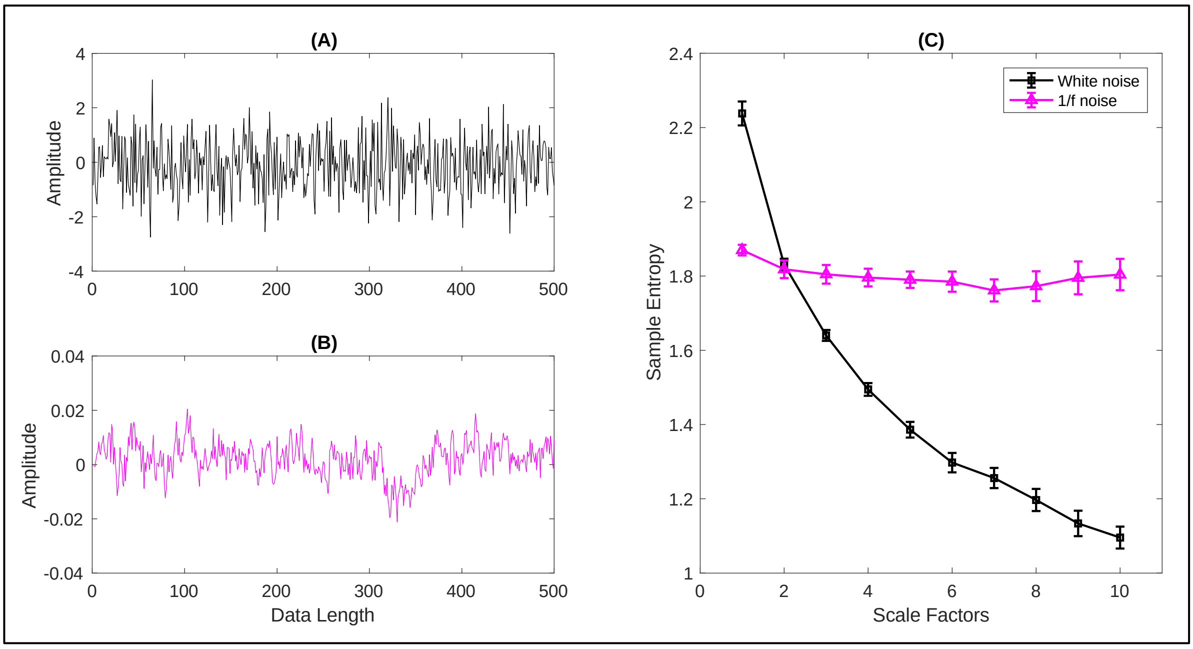

Table S1. Distributions, skewness and normality of blood pressure variability and complexity for the whole population, stratified by quintiles of age.

	SBP-CV				Complexity of SBP		
	n	Median (Interquartile Intervals)	Skewness	Normality	Median (Interquartile Intervals)	Skewness	Normality
**All**	908	4.7 (3.5 – 6.6)	2.66	<0.001	14.5 (13.2 – 15.7)	-0.89	<0.001
**Quintiles of Age:**							
**Q1 (<54.2)**	182	4.7 (3.5 – 5.9)	3.03	<0.001	15.1 (14.0 – 16.1)	-1.51	<0.001
**Q2 (54.2 – 64.7)**	181	4.3 (3.5 – 5.7)	2.74	<0.001	14.9 (13.6 – 16.1)	-0.95	<0.001
**Q3 (64.7 – 71.4)**	182	4.4 (3.3 – 5.9)	2.22	<0.001	14.3 (13.4 – 15.4)	-0.96	<0.001
**Q4 (71.4 – 77.7)**	181	5.3 (3.8 – 7.5)	1.49	<0.001	14.3 (12.9 – 15.6)	-1.02	<0.001
**Q5 (>77.7)**	182	5.3 (3.7 – 8.8)	2.32	<0.001	13.8 (12.4 – 15.1)	-0.29	0.253
	**DBP-CV**				**Complexity of DBP**		
	**n**	**Median (Interquartile Intervals)**	**Skewness**	**Normality**	**Median (Interquartile Intervals)**	**Skewness**	**Normality**
**All**	908	4.6 (3.3 – 6.6)	7.98	<0.001	14.7 (13.4 – 15.7)	-1.03	<0.001
**Quintiles of Age:**							
**Q1 (<54.2)**	182	4.6 (3.4 – 6.1)	4.29	<0.001	15.4 (14.1 – 16.4)	-1.69	<0.001
**Q2 (54.2 – 64.7)**	181	3.9 (3.1 – 5.5)	3.69	<0.001	15.2 (13.9 – 16.1)	-1.21	<0.001
**Q3 (64.7 – 71.4)**	182	4.1 (3.0 – 6.1)	2.86	<0.001	14.4 (13.2 – 15.4)	-0.65	<0.001
**Q4 (71.4 – 77.7)**	181	5.0 (3.6 – 7.1)	1.61	<0.001	14.4 (13.0 – 15.4)	-1.28	<0.001
**Q5 (>77.7)**	182	5.4 (3.7 – 8.6)	6.49	<0.001	14.0 (12.4 – 14.9)	-0.66	0.0016

Table S2. Distributions, skewness and normality of SBP-CV and SBP-Complexity, stratified by sex and by quintiles of age.

		SBP-CV			Complexity of SBP		
	n	Median (Interquartile Intervals)	Skewness	Normality	Median (Interquartile Intervals)	Skewness	Normality
**All Men**	497	4.6 (3.5 – 6.0)	3.85	<0.001	14.6 (13.4 – 15.9)	-0.42	<0.001
**Quintiles of Age:**							
**Q1 (<52.5)**	99	4.9 (3.6 – 6.0)	0.72	0.0018	15.0 (13.8 – 16.1)	-0.89	0.0004
**Q2 (52.5 – 62.9)**	100	4.3 (3.6 – 5.3)	1.87	<0.001	15.2 (13.8 – 16.1)	-0.45	0.057
**Q3 (62.9 – 70.7)**	99	4.0 (3.2 – 5.6)	2.48	<0.001	14.3 (13.4 – 15.7)	-0.21	0.12
**Q4 (70.7 – 77.5)**	100	4.9 (3.4 – 6.3)	1.98	<0.001	14.5 (13.5 – 15.9)	-0.40	0.029
**Q5 (>77.5)**	99	5.1 (3.9 – 7.5)	3.73	<0.001	14.3 (12.5 – 15.1)	-0.16	0.39
**All Women**	411	4.9 (3.5 – 7.7)	1.85	<0.001	14.5 (13.0 – 15.5)	-1.04	<0.001
**Quintiles of Age:**							
**Q1 (<56.3)**	82	4.4 (3.3 – 6.2)	2.73	<0.001	15.0 (13.8 – 16.2)	-1.63	<0.001
**Q2 (56.3 – 66.1)**	82	5.0 (3.4 – 7.1)	1.36	<0.001	14.8 (13.6 – 15.7)	-1.11	<0.001
**Q3 (66.1 – 72.3)**	83	4.6 (3.6 – 7.4)	1.82	<0.001	14.5 (13.2 – 15.2)	-1.25	<0.001
**Q4 (72.3 – 78.0)**	82	6.0 (4.0 – 8.1)	2.29	<0.001	14.2 (12.4 – 15.5)	-1.00	0.0007
**Q5 (>78.0)**	82	5.9 (3.4 – 10.5)	0.84	<0.001	13.6 (12.2 – 14.9)	-0.43	0.35

Table S3. **Distributions, skewness and normality of DBP-CV and DBP-Complexity, stratified by sex and by quintiles of age.**

	DBP-CV				DBP-Complexity		
	**n**	**Median (Interquartile Intervals)**	**Skewness**	**Normality**	**Median (Interquartile Intervals)**	**Skewness**	**Normality**
**All Men**	497	4.4 (3.2 – 6.1)	5.30	<0.001	14.7 (13.5 – 15.9)	-0.90	<0.001
**Quintiles of Age:**							
**Q1 (<52.5)**	99	4.7 (3.4 – 6.2)	2.65	<0.001	15.5 (14.0 – 16.4)	-1.91	<0.001
**Q2 (52.5 – 62.9)**	100	3.7 (3.0 – 5.0)	1.74	<0.001	15.3 (14.1 – 16.2)	-0.58	0.037
**Q3 (62.9 – 70.7)**	99	3.8 (2.9 – 5.7)	1.41	<0.001	14.3 (13.3 – 15.3)	-0.13	0.25
**Q4 (70.7 – 77.5)**	100	4.4 (3.3 – 5.9)	2.46	<0.001	14.8 (13.2 – 15.6)	-1.52	<0.001
**Q5 (>77.5)**	99	5.2 (3.8 – 7.6)	4.81	<0.001	14.1 (13.0 – 15.2)	-0.14	0.91
**All Women**	411	4.8 (3.4 – 7.2)	7.67	<0.001	14.7 (13.0 – 15.6)	-0.99	<0.001
**Quintiles of Age:**							
**Q1 (<56.3)**	82	4.3 (3.3 – 6.2)	3.47	<0.001	15.3 (14.0 – 16.4)	-1.47	<0.001
**Q2 (56.3 – 66.1)**	82	4.3 (3.2 – 6.3)	2.65	<0.001	15.2 (13.9 – 15.9)	-1.14	0.00012
**Q3 (66.1 – 72.3)**	83	4.5 (3.3 – 6.8)	2.66	<0.001	14.5 (13.0 – 15.3)	-0.17	0.01
**Q4 (72.3 – 78.0)**	82	5.6 (3.6 – 7.5)	7.70	<0.001	14.2 (12.3 – 15.5)	-1.34	<0.001
**Q5 (>78.0)**	82	5.6 (3.3 – 9.6)	1.27	<0.001	13.9 (12.2 – 14.8)	-0.79	0.01

Table S4. Correlates of blood pressure complexity, markers of autonomic function and vascular aging, unadjusted and adjusted for clinical variables.

		Complexity of SBP						Complexity of DBP					
		Un-adjusted		Adjusted (A+S)		Adjusted (A+S+RF)		Un-adjusted		Adjusted (A+S)		Adjusted (A+S+RF)	
	**n**	***r***	***p***	***Partial r***	***p***	***Partial r***	***p***	***r***	***p***	***Partial r***	***p***	***Partial r***	***p***
**HRV-SDRR**	**905**	0.20	<0.001	0.14	<0.001	0.12	<0.001	0.17	<0.001	0.11	<0.001	0.09	0.01
**HRV-RMSSD**	**905**	0.22	<0.001	0.17	<0.001	0.16	<0.001	0.18	<0.001	0.13	<0.001	0.11	<0.001
**BRS in LF**	**857**	0.19	<0.001	0.14	<0.001	0.13	<0.001	0.20	<0.001	0.15	<0.001	0.13	<0.001
**BRS in HF**	**824**	0.26	<0.001	0.21	<0.001	0.17	<0.001	0.26	<0.001	0.21	<0.001	0.17	<0.001
**SBP-PSD in LF**	**908**	-0.33	<0.001	-0.31	<0.001	-0.30	<0.001	-	-	-	-	-	-
**SBP-PSD in HF**	**908**	-0.0006	0.99*	0.004	0.91*	0.003	0.92*	-	-	-	-	-	-
**SBP-PSD LF/HF ratio**	**908**	-0.51	<0.001	-0.49	<0.001	-0.47	<0.001	-	-	-	-	-	-
**DBP-PSD in LF**	**908**	-	-	-	-	-	-	-0.30	<0.001	-0.30	<0.001	-0.30	<0.001
**DBP-PSD in HF**	**908**	-	-	-	-	-	-	-0.02	0.49*	-0.05	0.12*	-0.06	0.074*
**DBP-PSD LF/HF ratio**	**908**	-	-	-	-	-	-	-0.43	<0.001	-0.40	<0.001	-0.39	<0.001
**Pulse Pressure**	**908**	-0.14	<0.001	-0.05	0.15*	-0.02	0.56*	-0.13	<0.001	-0.04	0.25*	-0.01	0.70*
**PWV**	**815**	-0.21	<0.001	-0.11	0.001	-0.07	0.048	-0.21	<0.001	-0.11	0.0014	-0.07	0.055*

The association was determined by general linear model with a log-transformation. Three invalid quality of HRV recordings and those who do not meet the statistical criterion of BRS coherence were not included in the analysis of HRV and BRS. All analyses are statistically significant, except for analyses with a *. Adjusted (A+S), adjusted for age and sex; Adjusted (A+S+RF), adjusted for age, sex and cardiovascular risk factors of hypertension, diabetes, and smoking habit.

Figure S1. Distributions of complexity index and BPV, with Kernel-fitting curve. (A) SBP-CV; (B) DBP-CV; (C) SBP-complexity; and (D) DBP-complexity.

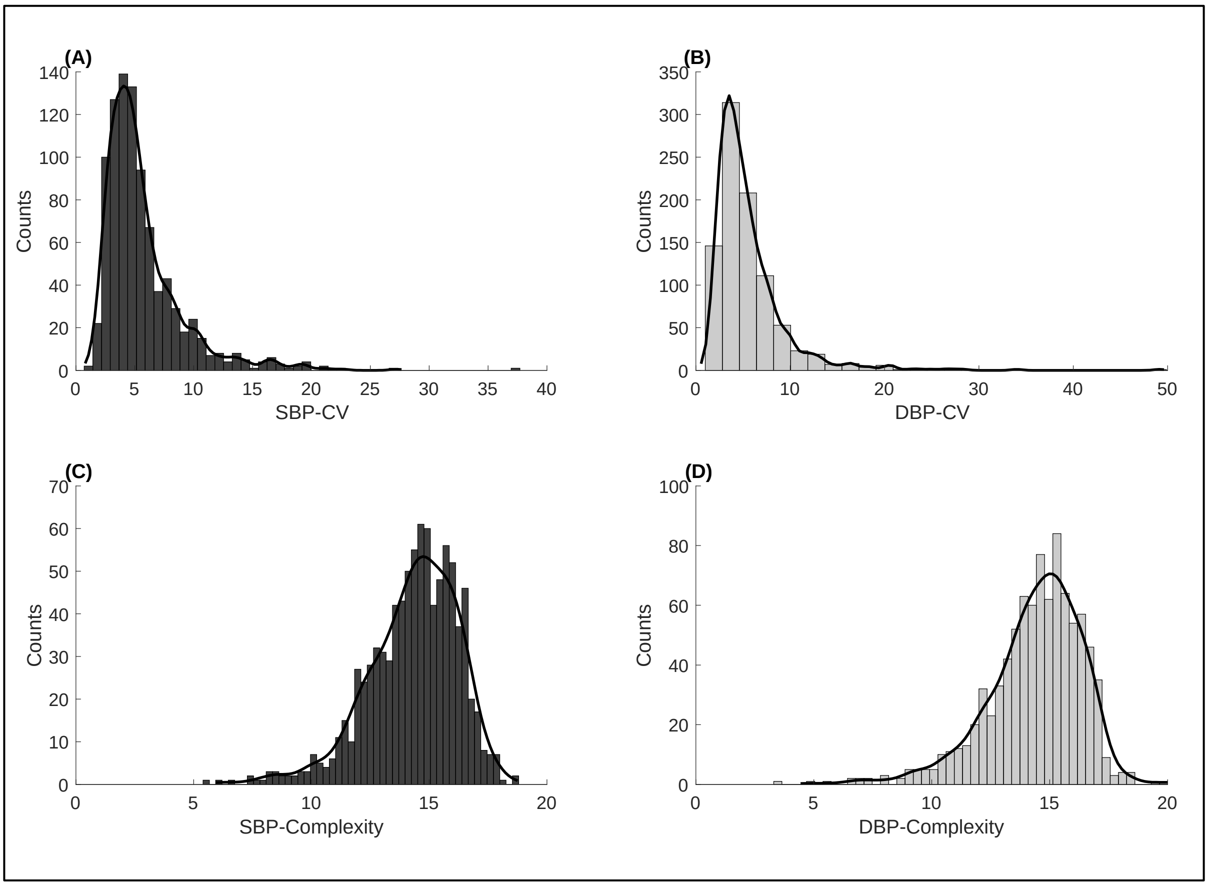

Figure S2. ModMSE curves of SBP and DBP, stratified by sex and by quintiles of age. (A – B) are modMSE of SBP and DBP in men, respectively; and (C – D) are modMSE of SBP and DBP in women, respectively.

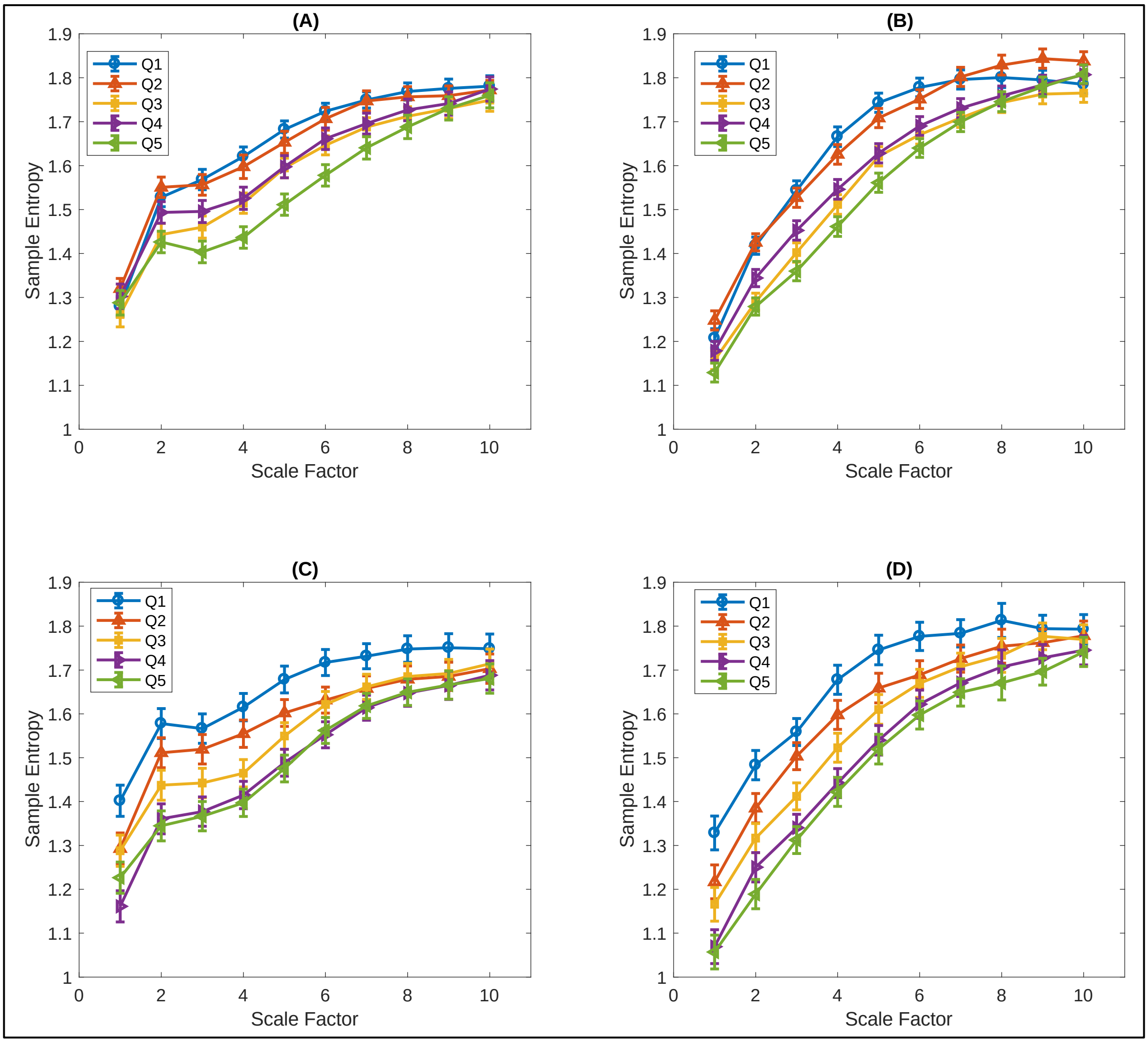

Figures are presented as mean ± standard error of the mean (SEM).

Figure S3. ModMSE curves of SBP and DBP, stratified by patients with TIAs and by strokes, suggesting that reverse causation is unlikely. (A – B) are modMSE of SBP and DBP in TIA patients, respectively; and (C – D) are modMSE of SBP and DBP in stroke patients, respectively.

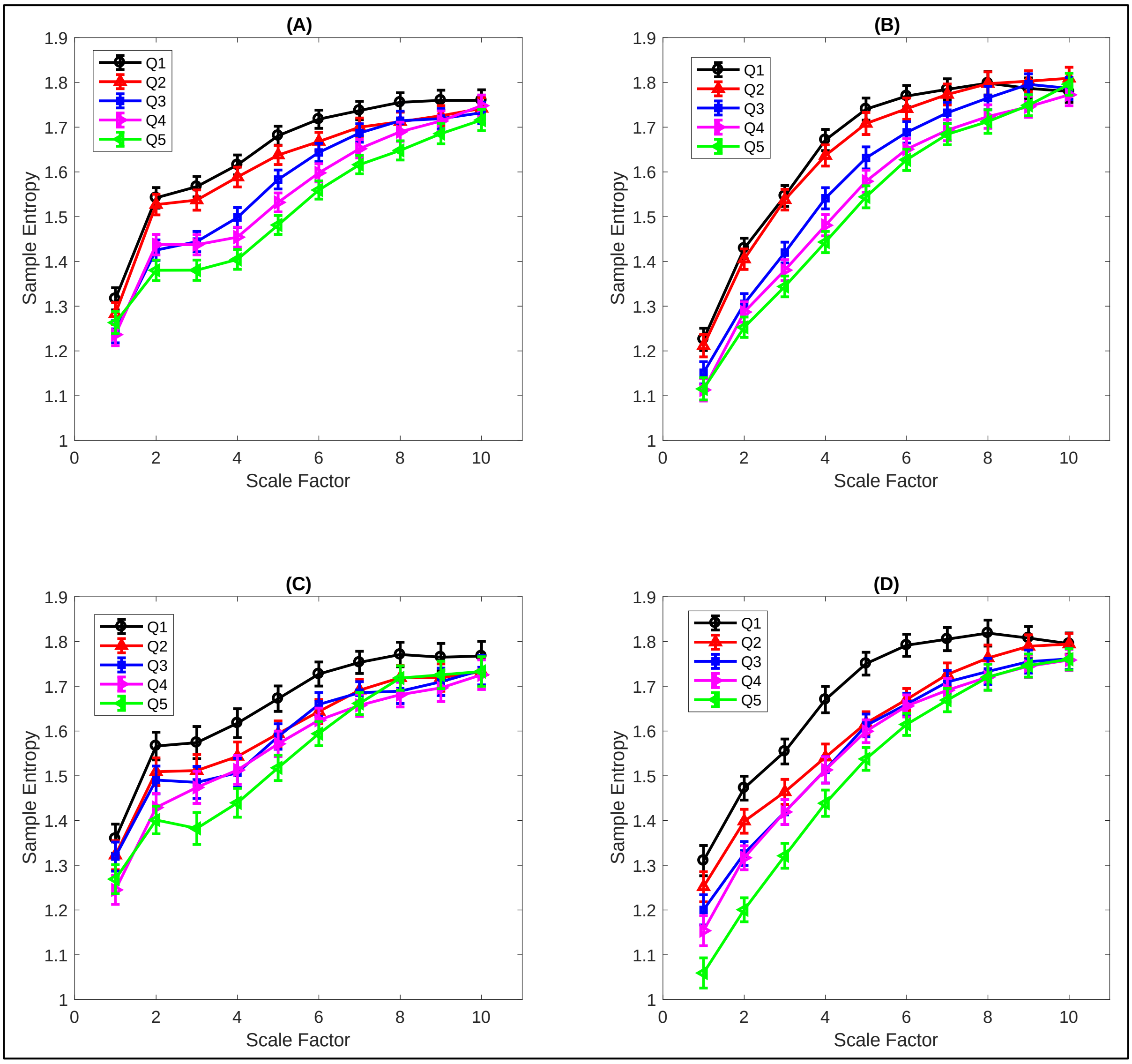

Figures are presented as mean ± standard error of the mean (SEM).

Figure S4. (A – B) The whole frequency spectrum for BRS-gain and SBP-PSD, stratified by high and low complexity of SBP (i.e. greater and lower than mean complexity values of SBP: 14.3 respectively); (C) the relationship between log-transformed SBP-complexity and SBP-PSD LF/HF ratio; (D – E) the whole frequency spectrum for BRS-gain and DBP-PSD, stratified by high and low complexity of DBP (mean DBP complexity: 14.4 respectively); and (F) the relationship between log-transformed DBP-complexity and DBP-PSD LF/HF ratio. Data are presented as mean ± standard error of the mean (SEM) and regression line with 95% CI.

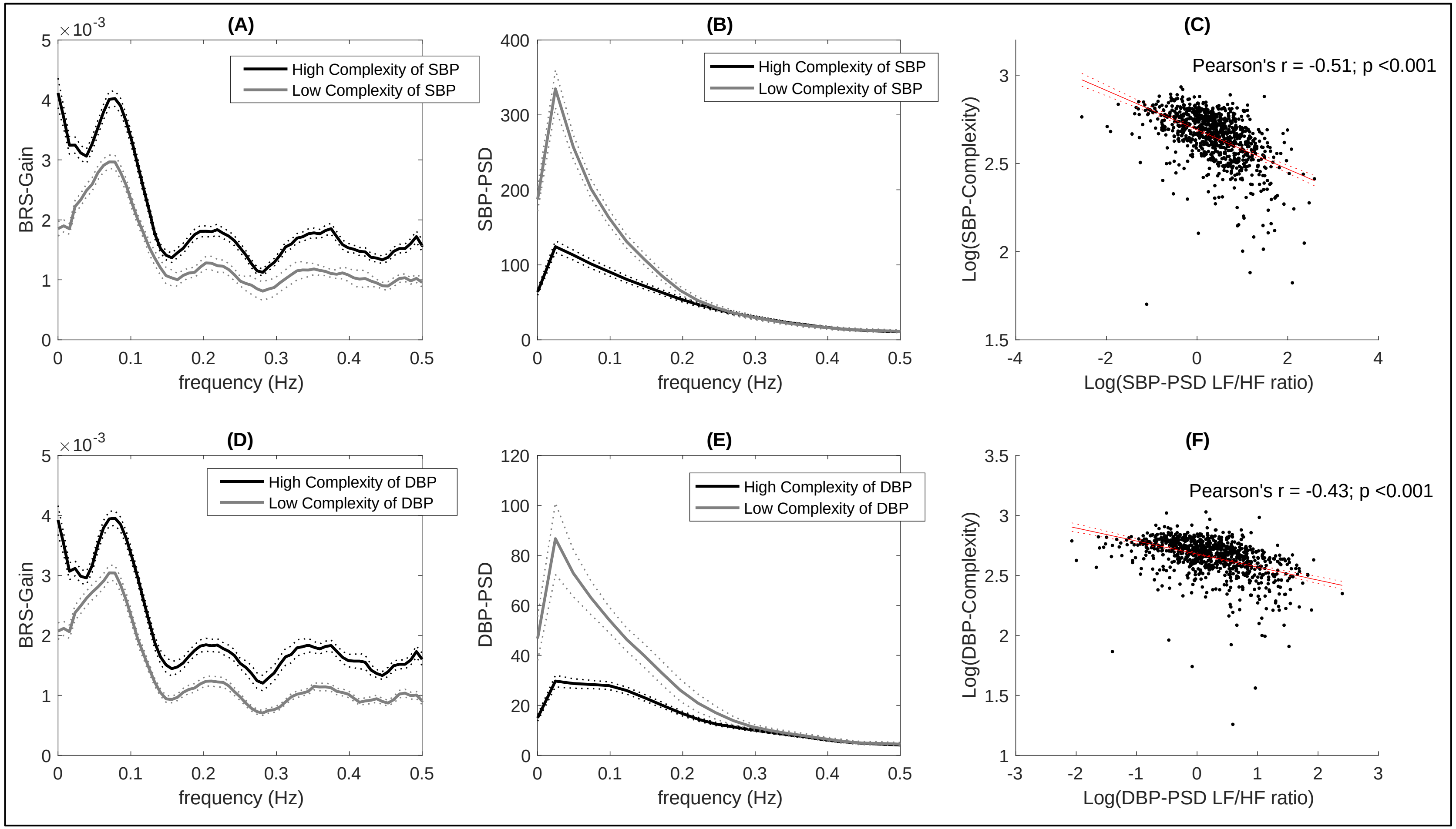

Figure S5. Values of complexity of DBP, stratified by quartiles of parameters of (A) SDRR and (B) RMSSD of R-R intervals; and BRS in (C) LF and (D) HF, respectively. Three invalid quality of HRV recordings and those do not meet the statistical criterion of BRS coherence were not included.

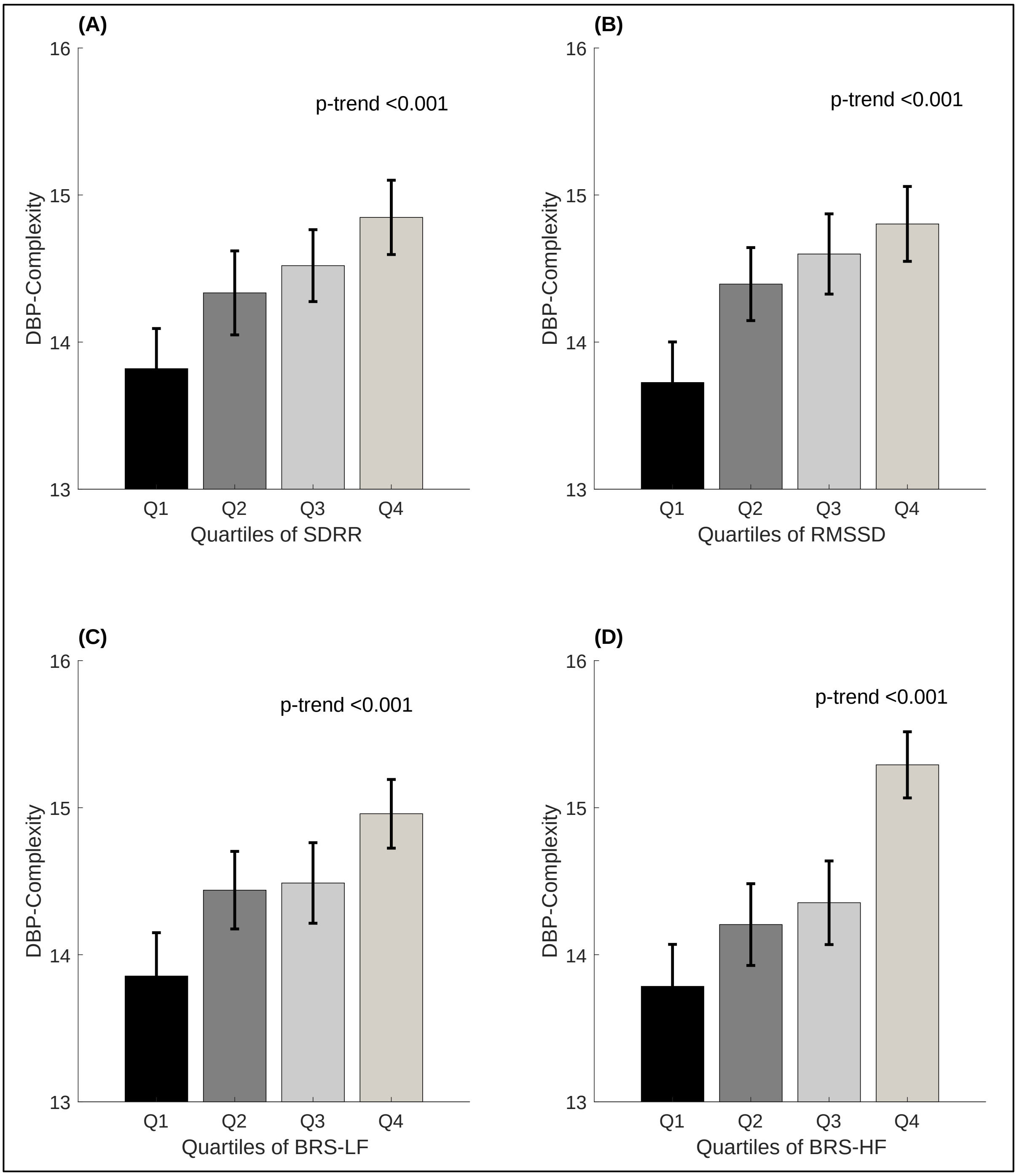

Data are presented as mean with 95% CI.

Figure S6. Complexity of DBP, stratified by quartiles of parameters of DBP-PSD in (A) LF and (B) HF bands, respectively; and (C) PP; (D) PWV.

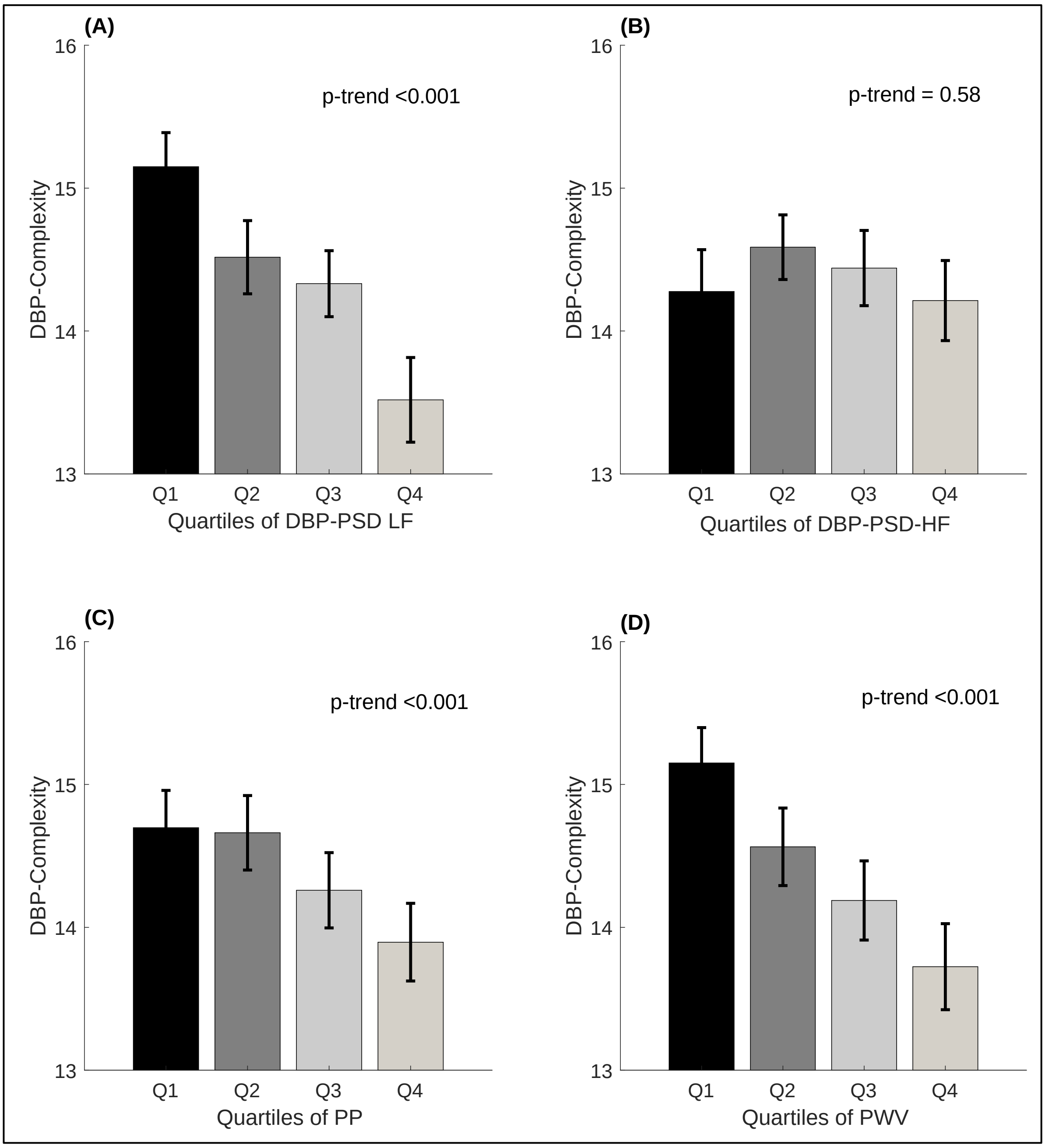

Data are presented as mean with 95% CI.
